# A novel machine learning methodology for the systematic extraction of chronic kidney disease comorbidities from abstracts

**DOI:** 10.3389/fdgth.2025.1495879

**Published:** 2025-02-04

**Authors:** Eszter Sághy, Mostafa Elsharkawy, Frank Moriarty, Sándor Kovács, István Wittmann, Antal Zemplényi

**Affiliations:** ^1^ Faculty of Pharmacy, University of Pécs, Pécs, Hungary; ^2^ Faculty of Sciences, University of Pécs, Pécs, Hungary; ^3^ School of Pharmacy and Biomolecular Sciences, Royal College of Surgeons in Ireland, Dublin, Ireland; ^4^ Medical School, University of Pécs, Pécs, Hungary; ^5^ Skaggs School of Pharmacy and Pharmaceutical Sciences, University of Colorado, Anschutz Medical Campus, Denver, CO, United States

**Keywords:** chronic kidney disease, comorbidities, systematic literature review, machine learning, active learning, named entity recognition, entity relation

## Abstract

**Background:**

Chronic Kidney Disease (CKD) is a global health concern and is frequently underdiagnosed due to its subtle initial symptoms, contributing to increasing morbidity and mortality. A comprehensive understanding of CKD comorbidities could lead to the identification of risk-groups, more effective treatment and improved patient outcomes. Our research presents a two-fold objective: developing an effective machine learning (ML) workflow for text classification and entity relation extraction and assembling a broad list of diseases influencing CKD development and progression.

**Methods:**

We analysed 39,680 abstracts with CKD in the title from the Embase library. Abstracts about a disease affecting CKD development and/or progression were selected by multiple ML classifiers trained on a human-labelled sample. The best classifier was further trained with active learning. Disease names in question were extracted from the selected abstracts using a novel entity relation extraction methodology. The resulting disease list and their corresponding abstracts were manually checked and a final disease list was created.

**Findings:**

The SVM model gave the best results and was chosen for further training with active learning. This optimised ML workflow enabled us to discern 68 comorbidities across 15 ICD-10 disease groups contributing to CKD progression or development. The reading of the ML-selected abstracts showed that some diseases have direct causal effect on CKD, while others, like schizophrenia, has indirect causal effect on CKD.

**Interpretation:**

These findings have the potential to guide future CKD investigations, by facilitating the inclusion of a broader array of comorbidities in CKD prognostic models. Ultimately, our study enhances understanding of prognostic comorbidities and supports clinical practice by enabling improved patient monitoring, preventive strategies, and early detection for individuals at higher CKD development or progression risk.

## 1 Introduction

Evidence-based medicine (EBM) provides a systematic approach to integrating the best available evidence, with clinical judgement and patient priorities, to inform clinical decision-making. EBM often relies on systematic reviews to synthesise clinical evidence, which play a critical role in updating clinical guidelines, optimising treatment strategies, and informing policy decisions, while the efficiency of these reviews is crucial in Health Technology Assessment (HTA) as it enables timely, and high-quality evidence synthesis to support informed decision-making on the adoption, reimbursement, and use of health technologies (1, 2). However, clinical evidence is primarily obtained from unstructured texts of scientific articles and the volume of published evidence in the medical field makes it increasingly difficult to keep up with the latest developments and to extract meaningful insights from research (3). Conducting manual literature reviews for systematic studies is not only labour-intensive and susceptible to human error, but also notably time-consuming, with the average estimated duration to complete and publish a systematic review being 15–16 months (4). The long and labour-intensive process of reviewing the literature does not only lead to delays in updating clinical guidelines but can also hinder the efficient synthesis of evidence for policy decisions such as HTA, pricing and reimbursement.

In recent years, machine learning (ML) algorithms have been explored as tools to expedite the systematic review process by automating text classification tasks (5–7). While promising, most existing ML approaches focus solely on identifying relevant articles, neglecting the extraction of entities (e.g., disease names) and the relationship between these entities (e.g., significant association between two diseases). This is a significant bottleneck when studying disease comorbidities, where determining the direction of relationships—such as identifying which disease contributes to the onset or progression of another—is crucial for developing clinical guidelines, identifying at-risk populations, and understanding the sequence of disease occurrence based on known patterns. Machine learning algorithms have the potential to aid in the review process by automating the text classification (8), information extraction (9), and entity relation extraction (10) tasks. These algorithms use statistical modelling to learn patterns in the data based on pre-labelled texts and can be trained to identify relevant information in medical literature.

Active learning is an approach used in machine learning to improve the efficiency of the training process. It involves selecting a subset of the available data for annotation by a human expert, with the aim of achieving the highest possible accuracy with the minimum amount of labelled data (11). This approach has shown to be effective in various machine learning applications such as image classification and bioinformatics (12).

Unlike large language models (LLMs), which are computationally intensive, ML models can be specifically optimised for text classification tasks, providing a lightweight and efficient alternative. This approach ensures scalability in resource-constrained environments while maintaining high performance. ML models also offer greater interpretability and customisation, enabling researchers to better understand and adapt them. Exploring these models can address practical challenges and facilitate hybrid systems that combine efficiency and accuracy with LLMs (13).

Chronic kidney disease (CKD) serves as an ideal case study to demonstrate the potential of these machine learning techniques, as it is a complex condition heavily influenced by comorbidities, where understanding the relationships between diseases is critical for improving early detection, prevention strategies, and patient management. CKD is a major public health issue worldwide, with increasing prevalence and associated morbidity and mortality (14). This debilitating condition often remains undiagnosed until the advanced stages due to its insidious nature and the absence of overt symptoms during the initial stages of deteriorating kidney function (15). The primary treatment modalities for CKD include conservative management to treat symptoms and prevent deterioration, and if it progresses, dialysis and kidney transplantation, with the costs of these therapies varying widely depending on the stage and severity of the disease. Dialysis, for instance, costs the National Health Service (NHS) around £30,800 per patient per year for haemodialysis and £23,500 per patient per year for peritoneal dialysis, based on data from 2010 (16). One factor that contributes to both the development of CKD and its progression is comorbidity, which is the presence of one or more additional diseases or conditions coexisting with CKD. The most common comorbidities associated with CKD include hypertension, diabetes mellitus, and cardiovascular disease, which are risk factors for its development and progression. However, recent studies have shown that other comorbidities, such as thyroid disorder, can also contribute to CKD pathogenesis and adversely affect clinical outcomes (17). Therefore, it is essential to acquire a greater understanding of these comorbidities and their interplay with CKD in order to optimise the management of this complex disease, and reduce its incidence and progression. Comprehensive knowledge of comorbidities can help clinicians provide more effective treatment and improve patient outcomes, which ultimately leads to a better quality of life for individuals living with CKD (18, 19).

Understanding the relationships between diseases within scientific abstracts is crucial for refining research focus in specific contexts. Rather than merely identifying diseases, extracting causal or consequential relationships enables researchers to discern how diseases interact, such as determining which conditions lead to specific comorbidities or identifying comorbidities resulting from a particular disease. In the context of CKD, which is associated with an extensive body of literature, narrowing the focus of a literature review to abstracts that explicitly address causal relationships between CKD and comorbidities helps filter out irrelevant studies and improves efficiency.

## 2 Research objective

The research objectives of this paper are twofold. Firstly, we aspire to develop an efficient machine learning workflow for text classification and entity relation extraction tasks, employed to systematically review and synthesise the literature. In doing so, we will address the difficulty of determining causal relationships between diseases, discerning which conditions lead to others. Secondly, our objective is to compile an extensive list of diseases that have been demonstrated to contribute to the development and/or progression of CKD, achieved through the review of abstracts from medical research articles. This comprehensive list will serve as a valuable resource for healthcare professionals, researchers, and policymakers to better understand and manage the interplay of conditions that precipitate chronic kidney disease. With this project, we also aim to emphasise the importance of comorbidities and the challenges associated with their identification in the context of clinical practice and research.

## 3 Methods

### 3.1 Data

Abstracts of articles related to chronic kidney disease were retrieved from the Embase library in March 31, 2022, with search terms “chronic kidney disease” or “chronic kidney failure” or “end stage renal disease” in the title, including title, author names, publication year and the link to the full text. 45,316 articles were found, of which 39,680 included abstracts and which were used in the analysis.

### 3.2 Disease named entity recognition

To eliminate abstracts that did not contain information about a disease impacting the development and/or progression of chronic kidney disease or its synonyms (referred to as CKD), our analysis commenced by identifying all disease terms within the abstracts and excluding those that mentioned CKD only. We utilised the NCBI disease corpus for disease named entity recognition, which comprised 793 PubMed abstracts and disease concept identifiers from MeSH or OMIM (20). Initially, the abstracts were cleaned by converting them to lowercase and removing special characters. Subsequently, a search was performed using the NCBI disease list on the abstracts. We also tested SpaCy's pre-trained disease named entity recognition model (en_ner_bc5cdr_md) (21) and compared the outcomes.

### 3.3 Manual labelling and text-preprocessing

Our initial objective involved the manual coding of a selected group of articles, specifically targeting those that discussed comorbidities of CKD. Our focus was narrowed to diseases contributing to the development and/or progression of CKD, deliberately omitting diseases that are consequent to CKD. In this process, we manually annotated 215 abstracts. Each abstract was assigned a binary value: “1” if it included information pertaining to a disease influencing CKD's development or progression, and “0” if it did not. This initial coding was crucial for setting a benchmark for our machine learning model's training. To maintain coding consistency, each abstract was independently coded by two researchers with relevant expertise. One of the researchers is a biostatistician with experience in data annotation and machine learning applications. The second researcher is an associate professor in pharmacy with extensive experience in population health and health services research. Any discrepancies in coding were subsequently addressed and reconciled through collaborative discussion.

All abstracts, including the pre-labelled ones, underwent pre-processing and vectorisation in preparation for the machine learning algorithms. The pre-processing steps included (i) converting to lowercase, (ii) eliminating whitespace, (iii) removing line breaks, (iv) removing leading labels, numbers, parenthetical expressions and URLs, (v) removing stopwords, (vi) discarding punctuation, (vii) tokenising, (viii) lemmatising, and (ix) applying word stemming. Following pre-processing, the abstracts were vectorised using the Term Frequency-Inverse Document Frequency (TF-IDF) method, where each vector initially included all unique words in the pre-processed corpus. To reduce dimensionality and sparsity, we restricted the vocabulary to the top 5,000 terms based on term frequency across the corpus, ensuring computational efficiency and maintaining classification performance.

### 3.4 Comparison of multiple machine learning classifiers

In the following step, we trained various machine learning (ML) algorithms on our manually labelled training dataset and validated them using the manually labelled test dataset. Our goal was to assign each abstract to one of two categories: 1 if the abstract includes information on a disease causing CKD development or progression, 0 otherwise. The classifiers were trained and validated using the manually labelled dataset of 215 abstracts and then applied to the full dataset of 35,109 abstracts.

We selected a diverse set of ML algorithms, including linear classifiers (SVM, SGD), ensemble methods (AdaBoost, Gradient Boosting, XGBoost, Random Forest), and a neural network classifier (MLP). Each method was chosen for its unique characteristics and potential advantages. Support Vector Machines (SVM) are known for their robustness and effectiveness in high-dimensional text data, they are well-suited for binary classification tasks like ours where the dataset is relatively small and features (words) are numerous (22). Stochastic Gradient Descent (SGD) was selected for its computational efficiency in optimising linear classifiers on sparse datasets, especially with TF-IDF vectorised text (22). Ensemble Methods were included due to their ability to combine the strengths of multiple decision trees, improving predictive performance and reducing overfitting. These methods are particularly effective at handling data with imbalanced classes (22). Finally, Multi-Layer Perceptron (MLP) method was included as it is capable of modelling nonlinear relationships in data and has shown success in various text classification tasks (22). The variety of classifiers allowed us to evaluate different modelling approaches and select the best-performing algorithm for this task, ensuring flexibility and robustness in the pipeline.

### 3.5 Evaluation and selection of the best classifier

**Figure 1:**
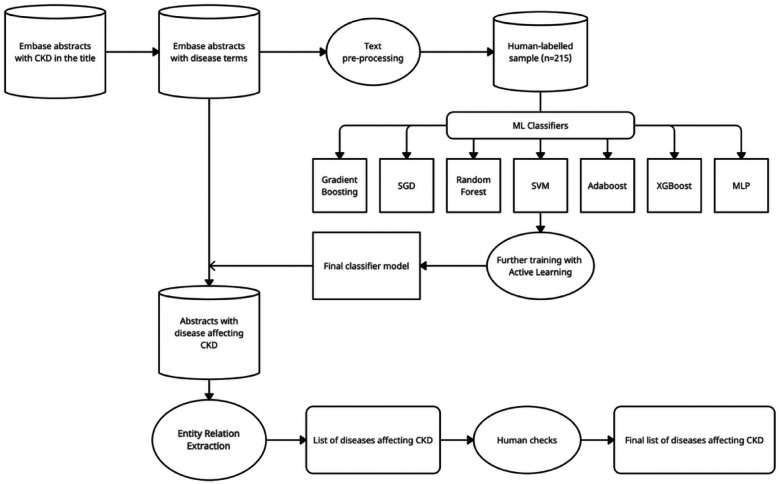
Machine learning pipeline.

We evaluated the performance of various ML algorithms by assessing their accuracy, precision, and F1 scores. To train the models, we randomly split the manually labelled dataset of *n* = 215 instances into training (70%) and testing (30%) sets. Prior to model training, we defined hyperparameters and their corresponding values for each algorithm using the GridSearchCV function. This function allowed us to determine the optimal hyperparameter configuration for each classifier algorithm based on their accuracy scores for both training and testing, as well as their F1 score and precision. We selected the best-performing model based on the F1 score (23), as different models exhibited varying levels of performance based on different metrics. The steps of the complete machine learning pipeline are visualised in Figure 1.

### 3.6 Active learning with SVC classifier

In this research phase, we employed the ActiveLearner modAL library to augment the training of our chosen model using the best-performing hyperparameters. Initially, we trained the active learner using the pre-coded 215 abstracts and then the trained active learner was applied to the unlabelled dataset to identify the most uncertain instances for further annotation. The original test set was not used during or after the active learning process to evaluate the model, as this would introduce bias. Using the predict_proba function and the max uncertainty sampling method, we identified unlabelled abstracts with the smallest probability score difference between label 1 and 0.

The active learning process was conducted iteratively, with 10 abstracts being manually labeled in each round. After each round of labelling, the newly annotated abstracts were added to the training set, and the model was retrained to incorporate the new data. The accuracy of the model was evaluated on the original testing set. After 6 rounds of labelling (a total of 60 abstracts), the model's accuracy had improved significantly from 0.85 to 0.94, and the probability score difference between label 1 and 0 increased, indicating that the decision boundary had stabilised. Based on these improvements, we determined that 60 abstracts were sufficient for the active learning phase. While the active learning process itself is automated, manual labelling of the selected abstracts was conducted by researchers, which limited the scale of the process.

To enable researchers to run the Python code on any computer console (in our case Terminal), we developed a function that allowed them to define the number of abstracts to label and create an Excel sheet containing a sample of abstracts ready for human labelling. After each round of labelling, the Excel sheet was saved, and the algorithm learned from the new labels. The labelling protocol employed in this phase mirrored that of the first “manual labelling” stage. Specifically, each abstract was assigned a binary value: “1” if it contained information related to a disease influencing the development or progression of CKD, and “0” if it did not. Consistent with our initial labelling approach, two researchers conducted this coding process independently to ensure accuracy and reliability.

### 3.7 Entity relation extraction

In this step, we encountered the challenge of identifying diseases mentioned in abstracts that were positively related to CKD, given that abstracts often mentioned multiple diseases with varying contextual relationships to CKD. To address this issue, we employed an entity relation extraction natural language processing (NLP) method that consisted of several steps. Firstly, we utilised the Spacy Stanza package's built-in negator function, coupled with a clinical negator termset (en_clinical), to exclude disease names negatively correlated with CKD. This step allowed us to eliminate those disease names that had no connection to CKD development or progression due to the negative correlation between the two. The resulting list was comprised of diseases that should not be neglected at this stage. Secondly, we applied different summarisation methods, including Gensim_summerize(), TextRankSummarizer(), LexRankSummarizer(), and LsaSummarizer(), to reduce the text size and eliminate irrelevant information. Since abstracts have varying wordings and structures, we utilised different summarisers in case the first one failed to provide a result that contained a disease name and CKD together. Finally, we employed Stanford OpenIE, a natural language processing AI, to identify disease names that had a positive relation to CKD. As OpenIE did not have a built-in clinical termset, it was programmed to yield results of only the positive relationship between disease names and CKD. In cases where OpenIE did not yield any results, we used a final dependency extractor NLP to find dependencies between CKD and disease names.

### 3.8 Python libraries used in the analysis

The analysis was conducted using several Python libraries to implement various components of the machine learning workflow and text extraction process. The Scikit-learn library (24) was central to our analysis, providing tools for data preprocessing, model training, hyperparameter optimisation, and evaluation. For active learning, we utilised the modAL library alongside Scikit-learn, supported by numpy, pickle, and atexit for efficient data handling and workflow management. Text extraction and natural language processing (NLP) tasks were performed using libraries such as stanza, spacy_stanza, negspacy, and pycorenlp for entity recognition and relationship extraction. Additional packages such as pandas, re, json, and collections were employed for data manipulation and preprocessing, while gensim and sumy were used for text summarisation. The nltk library supported feature extraction and preprocessing steps for machine learning. These libraries collectively enabled the streamlined execution of our machine learning pipeline, active learning framework, and NLP tasks.

### 3.9 Qualitative assessment of machine learning-selected abstracts

As a vital component of our methodology, the final stage involved a thorough qualitative review of the abstracts identified through our ML pipeline. This review process focused on a dataset comprising abstracts and other essential details such as author names, publication dates, links to the original articles, and the disease names identified within each abstract. Rather than reviewing all 35,109 abstracts, this step concentrated on the final list of cleaned and unique disease names extracted from the 2,954 abstracts labelled as relevant by the SVM classifier. At least one representative abstract was manually reviewed for each disease on the final list to confirm that it demonstrated a positive relationship with CKD.

The primary objective of this step was to verify whether the ML algorithms had accurately selected abstracts that provide information about diseases significantly impacting the development and/or progression of CKD. Specifically, our task was to filter out false positives—abstracts that either discussed diseases resulting as consequences of CKD, those with diseases not serving as significant predictors in models of CKD's development and/or progression, or diseases associated with conditions other than CKD. Given the diverse nature of statistical analysis language used across different abstracts, the potential for false positives in ML-assisted text reviews is notable. Therefore, this qualitative assessment phase was imperative to ensure the integrity of our results. Two researchers independently read and coded the abstracts, determining the appropriateness of their inclusion based on the study's criteria. Subsequently, we compared and reconciled these independent assessments to address any discrepancies in coding. This collaborative process culminated in the creation of a refined and accurate list of disease names associated with the development and/or progression of CKD, organised manually into ICD-10 disease categories.

## 4 Results

### 4.1 Manual labelling

In our initial manual labelling process, a sample of 215 abstracts was examined. Of these, 42 abstracts (19.53%) were coded as 1, signifying relevance to diseases affecting the development or progression of chronic kidney disease (CKD), while 173 abstracts (80.47%) were coded as 0, indicating no such relevance.

### 4.2 Disease named entity recognition

Utilising the SpaCy model initially resulted in several false positives, such as terms like “bleeding” or “microalbuminuria”. To refine our approach, we employed the NCBI disease list for more accurate entity recognition. Subsequently, 4,571 abstracts that only mentioned CKD and did not include other disease names were excluded. This refinement yielded a focused dataset of 35,109 abstracts, enhancing the efficacy of our semi-supervised machine learning algorithms and ensuring a more precise identification of relevant abstracts.

### 4.3 Machine learning classifier

Our comparative analysis of machine learning classifiers is detailed in Table 1. The SVM model demonstrated superior performance in terms of the F1 score (0.81). The table also shows that the best hyperparameter configuration for the SVM model proved to be setting penalty parameter (C) to 0.5, linear kernel, and the class weight to “balanced” due to the uneven distribution of the categories in the labelled sample. However, given its limited efficacy on the small manually labelled sample, we further refined the SVM model using active learning techniques with the same parameter settings.

**Table 1:** Results of ML classifiers on the labelled sample.

Model	Accuracy score (training)	Accuracy score (validation)	F1-score	Precision	Best parameters
Random Forest	0.91	0.83	0.77	0.71	{“class_weight”: “balanced”, “criterion”: “gini”, “max_samples”: 0.5, “n_estimators”: 10}
AdaBoost	0.89	0.82	0.78	0.76	{“algorithm”: “SAMME.R”, “learning_rate”: 0.5, “n_estimators”: 10}
Gradient Boosting	1.00	0.86	0.81	0.88	{“learning_rate”: 1.0, “loss”: “exponential”, “max_depth”: 4, “max_features”: 0.01, “min_samples_leaf”: 2, “n_estimators”: 1,000, “subsample”: 0.5}
SGD Classifier	1.00	0.82	0.78	0.76	{“alpha”: 0.0001, “class_weight”: “balanced”, “eta0”: 100, “l1_ratio”: 0.5, “learning_rate”: “adaptive”, “loss”: “log”, “max_iter”: 2,500, “penalty”: “l2”}
SVM	0.99	0.85	0.82	0.81	{“C”: 0.5, “class_weight”: “balanced”, “gamma”: 10, “kernel”: “linear”, “probability”: True}
XGBoost	1.00	0.82	0.78	0.76	{“learning_rate”: 0.5, “max_depth”: 5, “n_estimators”: 50}
MLPClassifier	1.00	0.80	0.80	0.79	{“activation”: “relu”, “alpha”: 0.0001, “hidden_layer_sizes”: 400, “learning_rate”: “constant”, “learning_rate_init”: 0.01, “solver”: “adam”}

### 4.4 Active learning classifier

The active learning phase involved labelling an additional 60 abstracts using the refined SVM algorithm. This process led to a notable increase in the average accuracy score of the classifier, from 0.85 to 0.94, and a Matthew's correlation coefficient of 0.85, indicating a strong alignment between predicted and actual values. As a result of this enhanced model, we successfully identified 2,954 abstracts that contained information relevant to diseases affecting CKD development or progression.

### 4.5 Qualitative assessment of abstracts and compilation of final disease list

**Figure 2:**
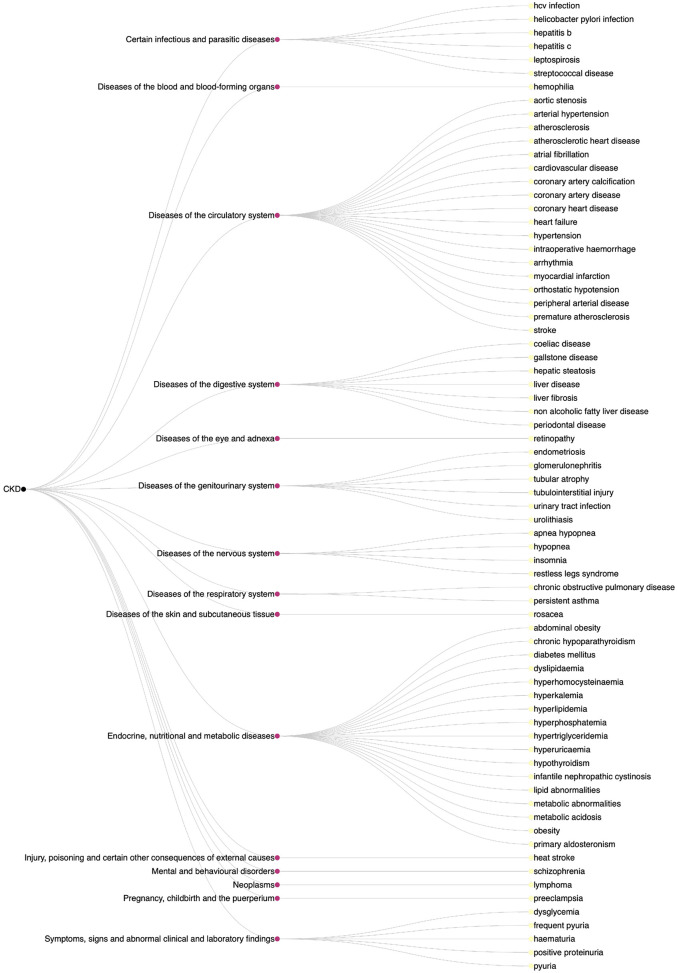
Dendrogram of diseases affecting the development and/or progression of CKD with ICD-10 disease categories.

The result of our study was the creation of a comprehensive disease list, comprising of 68 disease names across 15 ICD-10 disease categories, as shown in Figure 2. This list was refined by manually checking each disease name against the original abstracts to eliminate false positives, such as CKD complications (e.g., anaemia and abdominal aortic calcification) which were initially misidentified as causes.

## 5 Discussion

Our study has demonstrated an innovative methodology that employs machine learning to systematically identify comorbidities associated with chronic kidney disease. The current investigation emphasises the potential of utilising ML for literature review purposes. The various techniques detailed in this study possess the ability to discern not merely entities within texts but also the relationships and their orientations between these entities, as demonstrated by our identification of conditions relating to CKD development rather than CKD provoking other diseases. As this study focused on both development and progression, some identified comorbidities were consequences of CKD, however they were also related to progression to later stages of the disease. The method developed here could also be extended to differentiate studies focusing on CKD development and progression. This approach enabled us to extract sought-after entities from the texts, surpassing mere abstract classification.

Typically, studies may focus on a small number of comorbidities as risk factors of CKD, whereas this ML-assisted literature review discerned 68 comorbidities across 15 ICD-10 disease groups. These include less-known risk factors, such as rosacea (25) from the diseases of the skin and subcutaneous tissue group (L00-L99), insomnia (26) from the nervous system diseases group (G00-G99), and schizophrenia (27) from the mental and behavioural disorders group (F01-F99). This study identified a diverse range of comorbidities associated with either CKD development or progression, likely with different types of relationship. Some comorbidities have a direct causal effect on CKD development, for example well-characterised risk factors such as hypertension. Other identified comorbidities may have an indirect causal effect on CKD. For example, examining the primary literature, the association between schizophrenia and CKD was attributable to the antipsychotic medications prescribed to patients with schizophrenia.

To the best of our knowledge, no previous ML-oriented research has been conducted with the aim of extracting relationships between diseases from abstracts. Popoff et al. (7) performed a categorisation of medical abstracts into various disease domains utilising machine learning techniques. In their study, they compared three ML approaches (whereas our analysis involved seven) and executed classification based on both abstracts and complete texts, assessing the results through a range of accuracy metrics. Nonetheless, their methods were not employed for the extraction of information. Our approach has potential applicability in addressing other research applications that necessitate extensive review of a substantial volume of textual sources (e.g., abstracts) to comprehend the associations between potential predictors and a given disease. This methodology can be employed across various disease domains to investigate the described associations between comorbidities and specific diseases. Additionally, it could be utilised for discovering molecule-disease connections, thereby potentially enhancing the pace and efficacy of the drug development process. This method has been developed and identified a range of established and under-investigated comorbidities with prognostic value for CKD. However beyond this, the method can be extended to other disease areas and other domains, such as biomarkers, to enhance our understanding and accelerate scientific progress.

In the realm of healthcare policy, determining the value of various health technologies—ranging from pharmaceuticals to medical devices and procedures—is crucial for optimising the efficient use of scarce resources in healthcare. As health technology assessment requires evidence that is aligned with the specific context of the decision, and RCTs with the appropriate head-to-head comparisons are often not available, it is frequently required to conduct an extensive review to make indirect comparisons and network-metaanalyses (28).

The machine learning pipeline we developed, encompassing entity recognition, abstract categorisation, information extraction, and entity relation extraction, has significant potential for application in clinical research and HTA. One of its key strengths lies in the ability of ML algorithms to significantly speed up the study selection process in systematic literature reviews, which is vital in both EBM and HTA. Our methodology advances beyond mere literature categorisation. It includes entity relation extraction, a feature that not only identifies relationships between diseases, as demonstrated in our study, but can also discern links between health technologies and diseases, or among different health technologies. This aspect is particularly crucial in HTA and EBM, as it can greatly streamline the data collection process, enhancing the efficiency and depth of these reviews.

However, our approach is not without limitations. The constraints of this methodology include the necessity for human labelling during algorithm training and qualitative assessment of outcomes, as false positive results may emerge. These techniques are unsuitable for evaluating the quality of studies, hence alternative methods must be employed as part of systematic review and meta-analyses to critically appraise identified papers. Moreover, proficiency in Python programming is required, precluding execution via off-the-shelf, user-friendly platforms. Furthermore, the launch of the Generative Pre-trained Transformer (29) 4 in March 2023, a multimodal large language model (LLM) with enhanced AI capabilities, particularly in natural language processing, introduces a more efficient and accurate means for conducting systematic reviews and extracting disease relationships (29). This rapid advancement in AI technology underscores a challenge for researchers to stay current with the latest developments, positioning the use of prior ML technologies as a notable limitation of this study.

Traditional machine learning methods, such as Support Vector Machines (SVMs), were chosen for their robustness, interpretability, and ability to handle small to medium-sized datasets effectively. These methods are particularly well-suited for text classification tasks where transparency is critical, as in public health research. Alternative approaches, such as deep learning or LLMs, were not applied due to their high data requirements, lack of interpretability, and significant computational overhead, which were beyond the scope of this study. This rationale reflects the importance of traditional ML methods in maintaining transparency and reproducibility in public health research, especially given the limitations of LLMs. Nonetheless, the opacity of LLMs, often described as a “black box” leading to confabulation—where models generate fluent but factually incorrect information without a clear understanding of their processing—raises concerns (13, 30). While LLMs represent promising frontier, this opacity underscores the enduring value of traditional ML approaches that offer more interpretable and transparent workflows. This underlines the importance of maintaining traditional ML methods and a deeper comprehension of the internal workings of these models, which are integral to the “black box” of LLMs.

In summary, our research contributes to the evolving landscape of systematic reviews by integrating machine learning to enhance efficiency, transparency, and depth of analysis. While there are challenges and limitations, particularly in the context of data preparation and the need for human oversight, the potential benefits in terms of time-saving and the broadening of scope in systematic literature reviews are significant.

## Data Availability

The original contributions presented in the study are included in the article/Supplementary Material, further inquiries can be directed to the corresponding author.

## References

[1] Sackett DL Rosenberg WM Muir Gray JA Brian Haynes R Scott Richardson W. Evidence based medicine: what it is and what it isn't. Br Med J. (1996) 312(7023):71–2. 10.1136/bmj.312.7023.71 8555924 PMC2349778

[2] Guyatt G Cairns J Churchill D Cook D Haynes B Hirsh J Evidence-based medicine: a new approach to teaching the practice of medicine. JAMA. (1992) 268(17):2420–5. 10.1001/jama.1992.03490170092032 1404801

[3] Bastian H Glasziou P Chalmers I. Seventy-five trials and eleven systematic reviews a day: how will we ever keep up? PLoS Med. (2010) 7(9):e1000326. 10.1371/journal.pmed.1000326 20877712 PMC2943439

[4] Borah R Brown AW Capers PL Kaiser KA. Analysis of the time and workers needed to conduct systematic reviews of medical interventions using data from the PROSPERO registry. BMJ Open. (2017) 7:e012545. 10.1136/bmjopen-2016-012545 28242767 PMC5337708

[5] Zimmerman J Soler RE Lavinder J Murphy S Atkins C Hulbert L Iterative guided machine learning-assisted systematic literature reviews: a diabetes case study. Syst Rev. (2021) 10(1):97. 10.1186/s13643-021-01640-6 33810798 PMC8017891

[6] Marshall IJ Wallace BC. Toward systematic review automation: a practical guide to using machine learning tools in research synthesis. Syst Rev. (2019) 8(1):163. 10.1186/s13643-019-1074-9 31296265 PMC6621996

[7] Popoff E Besada M Jansen JP Cope S Kanters S. Aligning text mining and machine learning algorithms with best practices for study selection in systematic literature reviews. Syst Rev. (2020) 9(1):293. 10.1186/s13643-020-01520-5 33308292 PMC7734810

[8] Sebastiani F. Machine learning in automated text categorization. ACM Comput Surv. (2002) 34(1):1–47. 10.1145/505282.505283

[9] Moens M-F. Information Extraction: Algorithms and Prospects in a Retrieval Context. Vol. 21, 1. Aufl. ed. Dordrecht: Springer Netherlands (2006).

[10] Nasar Z Jaffry SW Malik MK. Named entity recognition and relation extraction: state-of-the-art. ACM Comput Surv. (2021) 54(1):20. 10.1145/3445965

[11] Settles B. Active Learning. Cham: Springer Nature Switzerland AG (2012).

[12] Shen R Yan K Tian K Jiang C Zhou K. Breast mass detection from the digitized x-ray mammograms based on the combination of deep active learning and self-paced learning. Future Gener Comput Syst. (2019) 101:668–79. 10.1016/j.future.2019.07.013

[13] Schwartz IS Link KE Daneshjou R Cortés-Penfield N. Black box warning: large language models and the future of infectious diseases consultation. Clin Infect Dis. (2024) 78(4):860–6. 10.1093/cid/ciad633 37971399 PMC11006107

[14] Bikbov B Purcell CA Levey AS Smith M Abdoli A Abebe M Global, regional, and national burden of chronic kidney disease, 1990–2017: a systematic analysis for the global burden of disease study 2017. Lancet. (2020) 395(10225):709–33. 10.1016/S0140-6736(20)30045-3 32061315 PMC7049905

[15] Couser WG Remuzzi G Mendis S Tonelli M. The contribution of chronic kidney disease to the global burden of major noncommunicable diseases. Kidney Int. (2011) 80(12):1258–70. 10.1038/ki.2011.368 21993585

[16] Kerr M Bray B Medcalf J O'Donoghue DJ Matthews B. Estimating the financial cost of chronic kidney disease to the NHS in England. Nephrol Dial Transplant. (2012) 27(Suppl 3):iii73–80. 10.1093/ndt/gfs269 22815543 PMC3484716

[17] Fraser SDS Roderick PJ May CR McIntyre N McIntyre C Fluck RJ The burden of comorbidity in people with chronic kidney disease stage 3: a cohort study. BMC Nephrol. (2015) 16(1):193. 10.1186/s12882-015-0189-z 26620131 PMC4666158

[18] Lee WC Lee YT Li LC Ng HY Kuo WH Lin PT The number of comorbidities predicts renal outcomes in patients with stage 3–5 chronic kidney disease. J Clin Med. (2018) 7(12):493. 10.3390/jcm7120493 30486496 PMC6306906

[19] Tonelli M Wiebe N Guthrie B James MT Quan H Fortin M Comorbidity as a driver of adverse outcomes in people with chronic kidney disease. Kidney Int. (2015) 88(4):859–66. 10.1038/ki.2015.228 26221754

[20] NCBI. The NCBI Disease Corpus (2023). Available online at: https://www.ncbi.nlm.nih.gov/research/bionlp/Data/disease/ (cited 2023).

[21] Zhang Y Zhang Y Qi P Manning CD Langlotz CP. Biomedical and clinical english model packages for the stanza python NLP library. J Am Med Inform Assoc. (2021) 28(9):1892–9. 10.1093/jamia/ocab090 34157094 PMC8363782

[22] Aggarwal CC. Machine Learning for Text. Cham: Springer International Publishing AG (2018).

[23] Galdi P Tagliaferri R Data mining: accuracy and error measures for classification and prediction. In: Ranganathan S Gribskov M Nakai K Schönbach C, editors. Encyclopedia of Bioinformatics and Computational Biology. Oxford: Academic Press (2019). p. 431–6.

[24] Pedregosa F Varoquaux G Gramfort A Michel V Thirion B Grisel O Scikit-learn: machine learning in python. J Mach Learn Res. (2011) 12:2825–30.

[25] Chiu H-Y Huang WY Ho CH Wang JJ Lin SJ Hsu YW Increased risk of chronic kidney disease in patients with rosacea: a nationwide population-based matched cohort study. PLoS One. (2017) 12(10):e0180446. 10.1371/journal.pone.0180446 28968402 PMC5624575

[26] Huang ST Lin CL Yu TM Yang TC Kao CH. Nonapnea sleep disorders and incident chronic kidney disease: a population-based retrospective cohort study. Medicine (United States). (2015) 94(4):e429. 10.1097/MD.0000000000000429 PMC4602952 25634175

[27] Wang H-Y Huang CL Feng IJ Tsuang HC. Second-generation antipsychotic medications and risk of chronic kidney disease in schizophrenia: population-based nested case–control study. BMJ Open. (2018) 8(5):e019868. 10.1136/bmjopen-2017-019868 29794090 PMC5988075

[28] Huang LY Werkö SS Merlin T Huang LY Schuller T. The ‘top 10’ challenges for health technology assessment: iNAHTA viewpoint. Int J Technol Assess Health Care. (2020) 36(1):1–4. 10.1017/S0266462319000825 31775943

[29] OpenAI. GPT-4 is OpenAI's most advanced system, producing safer and more useful responses (2023). Available online at: https://openai.com/gpt-4 (Accessed May 24, 2024).

[30] Editorial N. ChatGPT is a black box: how AI research can break it open. Nature. (2023) 619:671–2. 10.1038/d41586-023-02366-2 37491394

